# Partial ruptures of the distal triceps tendons show only slightly lower ultimate load to failure: a biomechanical study

**DOI:** 10.1186/s12891-023-06720-3

**Published:** 2023-07-19

**Authors:** Stephanie Geyer, Pavel Kadantsev, Daniel Bohnet, Christian Marx, Romed P. Vieider, Sepp Braun, Sebastian Siebenlist, Sebastian Lappen

**Affiliations:** 1 Department of Sports Orthopeadics, Technical University of Munich, Klinikum Rechts Der Isar, Ismaninger Straße 22, Munich, 81675 Germany; 2 St. Vinzenz Kliniken Pfronten Im Allgäu, Pfronten, Germany; 3 UMIT - Private Universität für Gesundheitswissenschaften, Medizinische Informatik und Technik, Private University Hall, Hall/Tirol, Austria; 4 Gelenkpunkt - Sports and Joint Surgery Innsbruck, Innsbruck, Austria

**Keywords:** Distal triceps tendon, Tendon rupture, Rupture mechanism, Partial ruptures, Biomechanics

## Abstract

**Objective:**

Partial ruptures of the distal triceps tendon are usually treated surgically from a size of > 50% tendon involvement. The aim of this study was to compare the ultimate load to failure of intact triceps tendons with partially ruptured tendons and describe the rupture mechanism.

**Methods:**

Eighteen human fresh-frozen cadaveric elbow specimens were randomly assigned to two groups with either an intact distal triceps tendon or with a simulated partial rupture of 50% of the tendon. A continuous traction on the distal triceps tendon was applied to provoke a complete tendon rupture. The maximum required ultimate load to failure of the tendon in N was measured. In addition, video recordings of the ruptures of the intact tendons were performed and analysed by two independent investigators.

**Results:**

A median ultimate load to failure of 1,390 N (range Q_0.25_—Q_0.75,_ 954 – 2,360) was measured in intact distal triceps tendons. The median ultimate load to failure of the partially ruptured tendons was 1,330 N (range Q_0.25_—Q_0.75,_ 1,130 – 1.470 N). The differences were not significant. All recorded ruptures began in the superficial tendon portion, and seven out of nine tendons in the lateral tendon portion.

**Discussion:**

Partial ruptures of the distal triceps tendon demonstrate a not statistically significant lower ultimate load to failure than intact tendons and typically occur in the superficial, lateral portion of the tendon. This finding can be helpful when deciding between surgical and conservative therapy for partial ruptures of the distal triceps tendon.

## Background

Triceps tendon ruptures are rare injuries and occur most often in middle-aged, physically active men [1, 2] with partial tendon ruptures representing more than 50% of the cases [3]. Due to their rarity, the injury mechanism of the distal triceps tendon is poorly understood. According to Lee et al. [4], a fall on an outstretched arm typically results in partial tendon ruptures. These partial ruptures were observed to primarily occur in the long and lateral head leaving the medial head uninjured. In a radiological study, the superficial portion of the tendons was described to be primarily involved in partial tendon ruptures [5]. However, the assessment of the distal triceps tendon in MRI is highly prone to errors and often does not match the intraoperative findings [6].

Further, indication for surgical treatment of partial ruptures is still controversially discussed. Although not based on scientific data, a rupture size of > 50% of the tendon is often given as an indication for surgical treatment [1]. In contrast, multiple successful reports of non-operative treatment of partial ruptures have been described in the literature [7–11]. Subsequently, this raises the question of whether non-operative treatment for partial distal triceps tendon ruptures might be more favorable in these cases than previously presumed. While the ultimate load to failure of intact triceps tendons has previously been determined [12], no biomechanical data is currently available regarding the ultimate load to failure of partially ruptured tendons.

The purpose of this study was to determine whether the ultimate load to failure of partially ruptured and intact triceps tendons differ significantly, and further to describe the rupture mechanism. The hypothesis was that there will be no significant differences in ultimate load to failure and that the superficial tendon fibers would rupture primarily.

## Methods

**Fig. 1:**
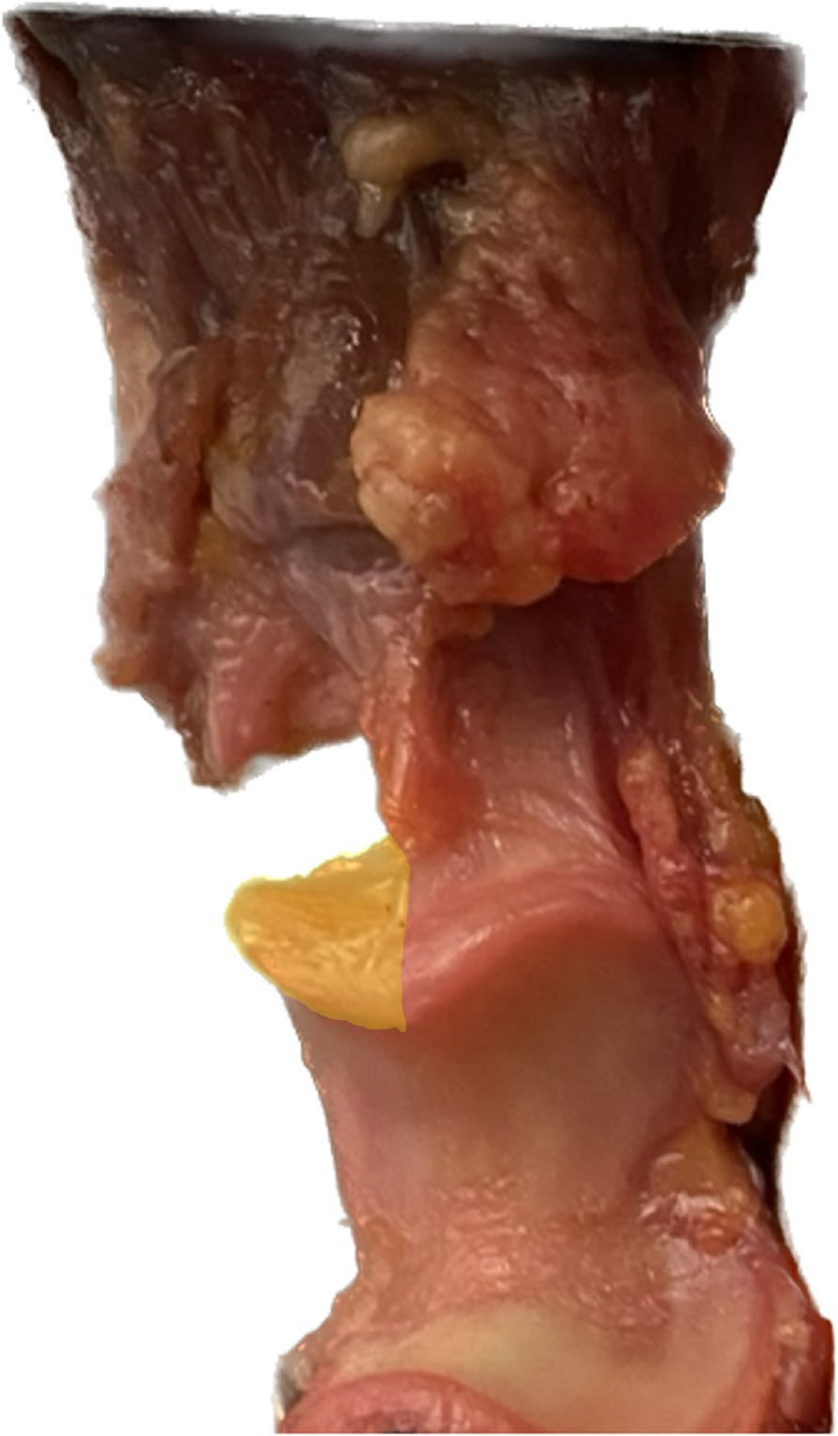
The incision of the distal triceps tendon was cut laterally to simulate a partial tendon rupture of 50% (marked area). Both superficial as well as deep tendon fibres were severed

Institutional review board approval was obtained prior to commencement of this study (2022–436-S-SR). Eighteen fresh-frozen cadaver elbows with no gross evidence of tendon injuries, ligament tears, fractures or bony deformities were used in the present study after two specimens had to be excluded due to large olecranon fractures occurring in pilot testing due to poor bone quality. The specimens were thawed for 24 h preceding dissection and testing. Specimens were dissected free of skin and subcutaneous tissue. All soft tissue was removed except for the distal triceps tendon and the triceps brachii muscle. The elbows were exarticulated and only the ulna with the triceps attached were used for testing. The specimens were then randomized to an intact tendon group and a partially ruptured tendon group. For the partially ruptured tendon group, the bony insertion points of the distal triceps tendon were measured using calipers. The size of the footprint was then calculated and 50% of the tendon was cut laterally with a scalpel at the bony attachment including both superficial and deep tendon fibers (Fig. 1).

### Biomechanical testing

**Fig. 2:**
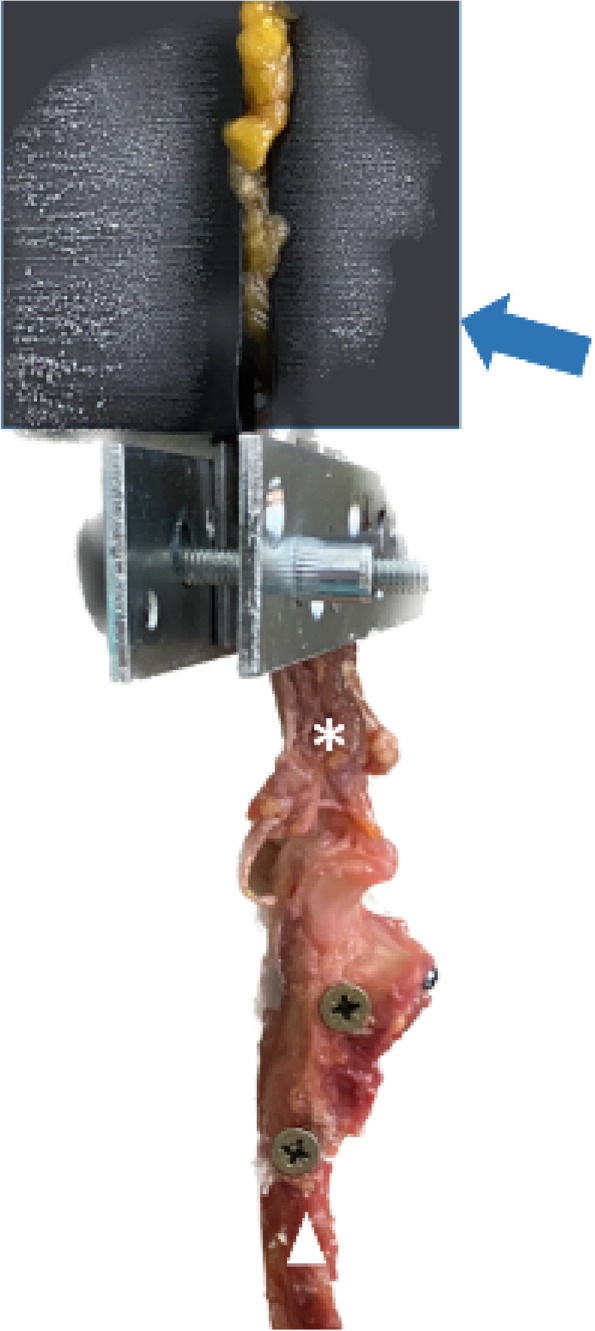
The triceps muscle and tendon (star) were fixed using cryoclamps (blue arrow) with the ulna (triangle) positioned in full elbow extension in relation to the distal triceps tendon

The prepared specimens were fixed to a custom jig and secured to the actuator of the universal tensile testing machines (Z010 Test Controll II, ZwickRoell, Ulm, Germany). The triceps muscles of the prepared specimens were then fixed using cryoclamps in a way that all fibers of the triceps tendon were stretched as evenly as possible. The ulna was positioned in accordance with the experimental design by Petre et al. [12] allowing unidirectional tensile force (Fig. 2).

A continuous pull of 30 mm per minute applied to the tendon until the tendon ruptured completely was determined in pilot testing in order to best observe the rupture mechanism. The ultimate load to failure (N) was measured for each specimen. Specimen were excluded if bony avulsion of the tendon occurred.

### Video analysis

The ruptures of the intact tendon group were filmed from a radial and posterior view in order to analyze the rupture mechanism and determine which fibers would rupture primarily in an uninjured tendon. The videos were then independently evaluated by two investigators (S.L. and D.B.). A third examiner (P.K.) was consulted to independently examine videos if the first two investigators disagreed.

### Statistical analysis

The sample size was calculated using the G*Power software (latest version 3.1.9.7; Heinrich Heine Universität Düsseldorf, Düsseldorf, Germany). Using the effect size in terms of peak failure load, referring to data previously published by Scheiderer et al. [13] determining difference between two independent means to calculate sample size. α was set at 0.05. The total samples size of 18 divided into 9 per group was required to achieve power of 0.95.

All calculations were performed with SPSS Statistics (Version 28, Property IBM Corp., NY, USA). Descriptive statistics were used for continuous variables. Normal distribution was assessed by the Shapiro–Wilk test. Normally distributed values were described by mean and standard deviation, skewed distributed values by median and interquartile range (range Q_0.25_—Q_0.75_). Paired t-tests (for normally distributed data) and Wilcoxon-tests (for non-normally distributed data) were used to assess differences between the intact and partially ruptured tendons. A value of *p* < 0.05 was considered significant.

## Results

The intact tendon group consisted of six left and three right elbows from six male and three female cadavers with a median age of 79.0 years (range Q_0.25_—Q_0.75_, 73.0 – 88.0 years). The partially ruptured tendon group was made up from one left and eight right elbow joints from six male and three female cadavers with a median age of 79.0 years (range Q_0.25_—Q_0.75_, 72.0 – 87.0 years).

**Fig. 3:**
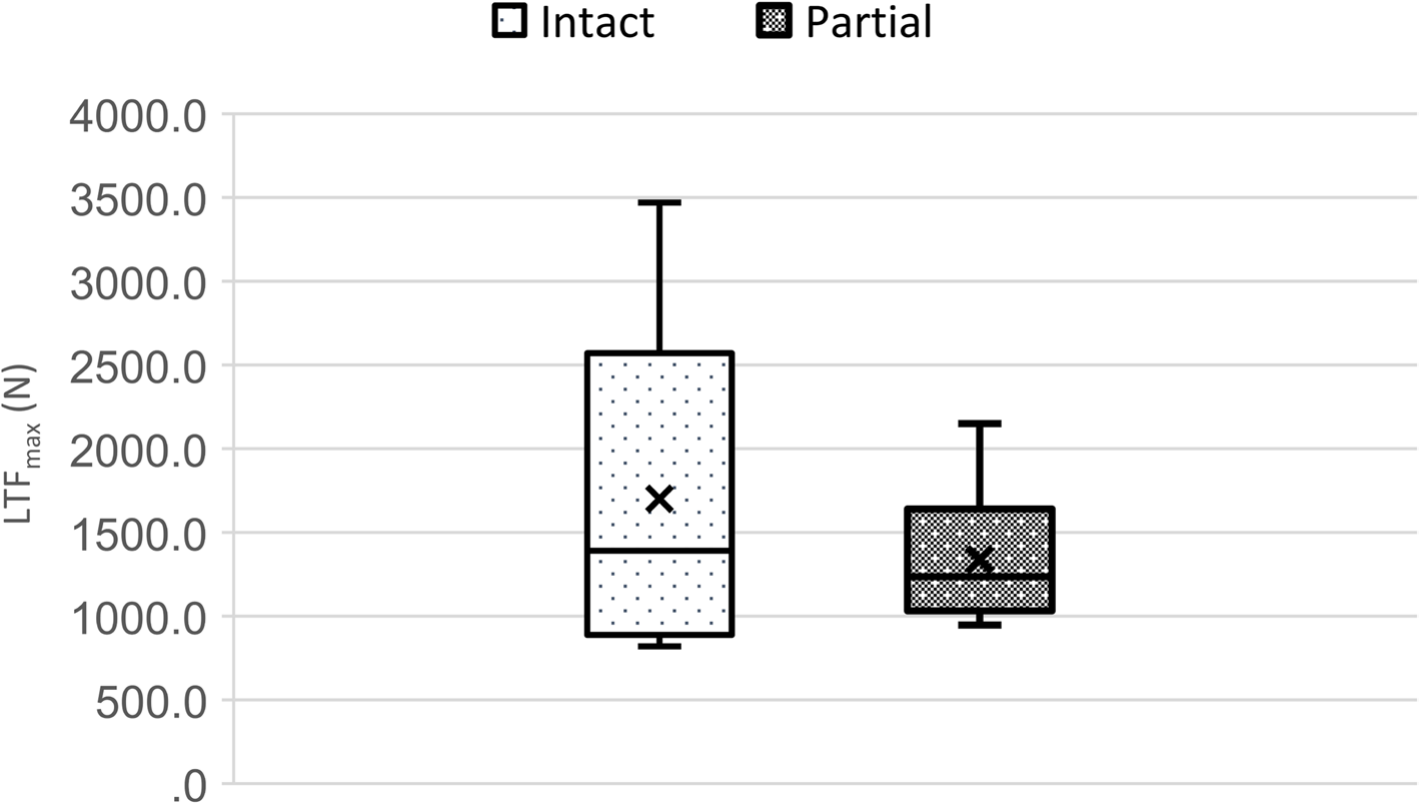
Boxplot diagram of maximum load to failure (LTF_max_) in Newton (N) of intact vs. partially ruptured triceps tendons

The median ultimate load to failure of the intact tendon group was 1,390.0 N (range Q_0.25_—Q_0.75,_ 954.0 – 2,360.0) and 1,330.0 N (range Q_0.25_—Q_0.75,_ 1,130.0 – 1.470.0 N) for the partially ruptured tendon, respectively (Fig. 3). The differences between the groups showed no statistical significance.

**Fig. 4:**
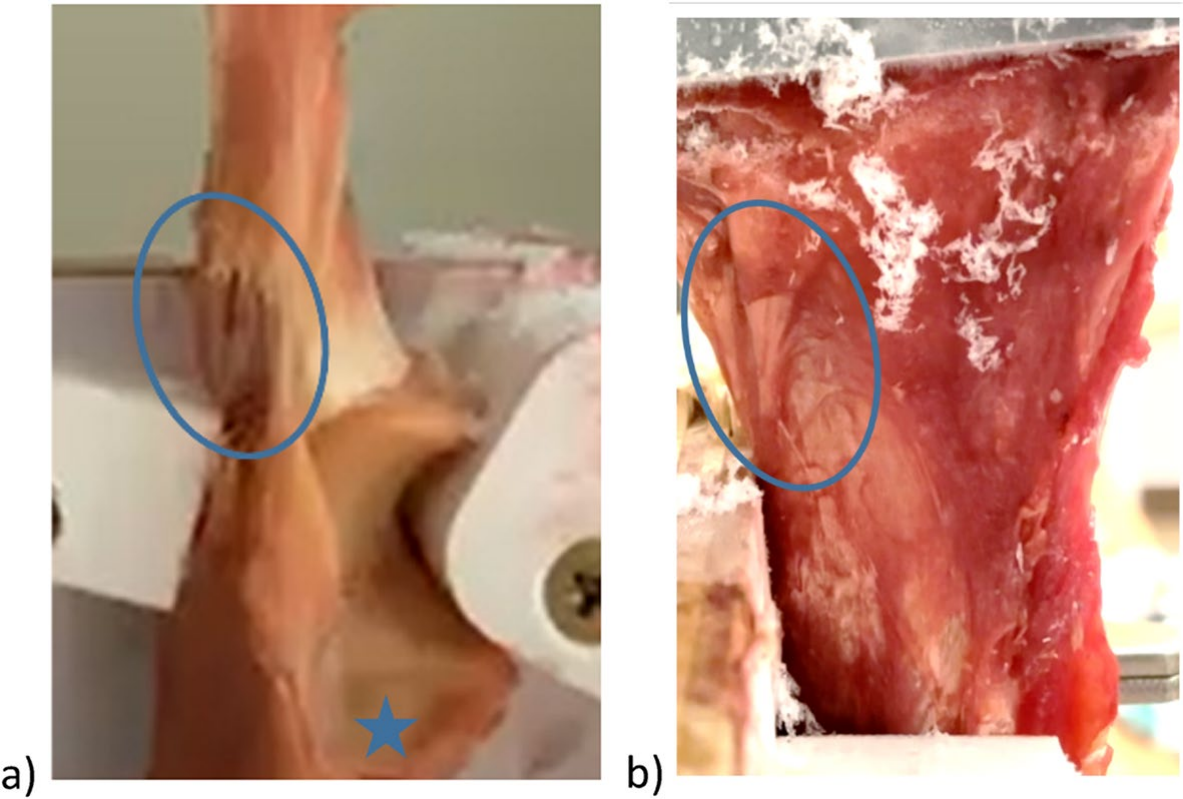
The rupture process of the intact distal triceps tendons was observed from radial (**a**) and posterior (**b**). The rupture was seen most common starting in the superficial and lateral tendon fibres. (Star: radial notch; circle: torn tendon fibres)

Agreement between the investigators could be reached for all videos. The analysis showed that the tendon rupture started in the superficial tendon fibers in all cases and in the lateral tendon fibers in seven of nine cases (Fig. 4a and b).

In one case the rupture originated medially and in one case it originated centrally and extended simultaneously medially and laterally.

## Discussion

The most important finding of the present study is that although partial ruptures of distal triceps tendons tend to withstand a lower ultimate load to failure in comparison to intact tendons, this difference was not statistically significant. The second finding is that distal triceps tendon ruptures begin most often in the superficial and lateral tendon fibers.

Petre et al. [12] first determined that the average ultimate load to failure of intact distal triceps tendons was 1,741 N. The present study found comparable results with a median ultimate load to failure of 1,390 N. Interestingly, significantly lower ultimate load to failure of 234—737 N are described in the literature for repaired distal triceps tendons [12–15]. At a median of 1,300 N, the ultimate load to failure determined in this study for partially torn tendons was therefore significantly closer to the ultimate load to failure of intact tendons than to those of repaired tendons. Distal triceps tendons with a 50% partial rupture thus show better primary stability than repaired tendons. However, an in-depth comparison to repaired tendons, especially of partial ruptures, should be further investigated in the future.

Interestingly, there was greater dispersion of failure loads in the intact tendon group than in the partially ruptured tendon group. This could be due to the limited size of the groups and the fact that no matched pairs were available for this study. Although the groups were comparable in age and sex, other characteristics such as bone and tendon quality may have varied. It is also possible that the failure loads correlate with the tendon diameter. Since the overall diameter of the intact tendons is larger and therefore probably more dispersed than that of the partially ruptured tendons, it is possible that this effect carries over to the failure loads. However, the correlation between tendon diameter and failure load needs to be further investigated.

In the current literature, surgical management is recommended for partial tears affecting more than 50% of the tendon [1, 16]. However, reports of non-operatively treated distal triceps tendons are rare. Harris et al. [17] report the conservative treatment of a 39-year-old patient with bilateral partial triceps tendon ruptures of 50% and 70%. The patient returned to weightlifting four weeks after his injury and recovered to near-normal function at 41 weeks. Similar reports confirm these promising results in patients with high functional demands [7–11]. However, there are also less successful reports. In a case series by van Riet et al. [18], 60% of conservatively treated patients with partial triceps ruptures showed limited to no improvement, and 40% required reconstruction over time. Mair et al. [19] also reported that in American football players who were treated initially conservatively some suffered complete tendon ruptures and underwent subsequent surgery. Since no significant difference was seen in the ultimate load to failure of intact and partially ruptured tendons, the data collected in this study suggests that conservative therapy could be successful in cases of partial ruptures larger than 50% of the tendon. This argument can be made in particular because the ultimate load to failure of repaired tendons described in the literature are significantly lower than those found in the present study for 50% partially torn tendons [12–15]. However, these are time-zero failure loads and cannot account for healing of the repaired tendon or possible progress of the partially ruptured tendon. Further studies need to be conducted to determine at what size a partially torn tendon shows a significant difference from intact tendons and at what point its ultimate load to failure approach those of repaired tendons. It therefore remains difficult to make a definitive decision on when to decide for or against surgery based on the data currently available. Thus, a variety of factors should influence the decision-making process for partial ruptures, including the patient's pain, residual strength for elbow extension, comorbidities, the patient's age, and the patient's expectations and lifestyle [16]. However, based on the present data, it can be argued that partial tears affecting less than 50% of the tendon might best be primarily treated nonoperatively as the ultimate load to failure measured in partially ruptured tendons were not significantly lower compared to fully intact tendons. In cases of larger partial ruptures, both conservative and surgical therapies should be discussed to the patient. Moreover, patients with less affected extension strength might be more likely to succeed nonoperatively, while physically very active patients may benefit from surgical treatment.

Based on radiological observations, Lee et al. [4] described, that partial ruptures of the distal triceps tendon typically occur in the superficial part of the distal triceps tendon. The anatomy of the distal triceps tendon insertion at the olecranon offers a possible explanation. Several studies have shown that the medial head tendon is distinct from a common tendon of the long and lateral head: while the long and lateral heads combine to form a superficial tendon inserting at the olecranon process, the medial head courses below this tendon to its insertion at the olecranon [20, 21]. Lee et al. [4] hypothesized that the lateral and long heads of the triceps muscle play a primary role in eccentric contractures in falls from outstretched arm injuries resulting in exclusively superficial tendon ruptures. In the present biomechanical model, the superficial tendon was also observed to rupture before the deep tendon of the medial head. This suggests that not only the mechanism of the injury is responsible for the fact that the tendon of medial head usually remains intact in partial ruptures, but also that the rupture pattern is caused by different resistances of the different tendon parts themselves. Knowledge of this may also be helpful in diagnostic means: while MRI has been shown to be sensitive in diagnosing distal triceps tendon ruptures, it has also been shown to be imprecise in distinguishing between full and partial tears. This can lead to false-positive interpretation of a partial tendon rupture.

This study demonstrates that the lateral tendon structures tend to tear before the medial ones. This is somewhat in contrast to reports of medial partial tendon ruptures with simultaneous involvement of the medial ligament complex [22]. However, it has to be clearly stated, with these injuries typically resulting from a fall onto the outstretched arm, an additional valgus load onto the elbow is exerted. This effect was not reproduced in the present study.

Several limitations of this study must be acknowledged. This includes the inherent limitations of in vitro studies. The tests were performed on cadaveric specimens and at time point zero, which fail to simulate in vivo conditions, including biological healing and tissue regeneration. This study is also limited by its small sample size, which, however, is similar to that of other biomechanical studies on distal triceps tendon ruptures [13, 14, 23]. Even though an a priori power analysis had been performed, it is possible that the wide range observed in the intact triceps tendon group is due to the study being underpowered and a larger sample size might have reduced this effect. However, its’ mean value of 1.300 N is comparable to values previously described [12] and are therefore likely to be reliable. In addition, no matched pairs of elbows were available for testing, which would have led to less confounding effects. However, the groups did not differ in gender distribution and only slightly in age. Furthermore, the age of the specimens was significantly older than the average age of patients with distal triceps tendon ruptures. However, biomechanical studies such as the present often have to use specimens that are older than the average patient, as the average body donor is significantly older. Distal triceps tendon ruptures often occur based on tendinopathy, which is thought of to be predisposing factor [24]. Therefore, it is possible that specimens from donors suffering from tendinopathy may require less ultimate load to failure. However, since tendinopathies of the distal triceps tendon are rare, this factor probably can be neglected. Other predisposing factors such as steroid injections or predisposing internal diseases were also not known. The biomechanical set-up was chosen in accordance with Petre et al. to allow for comparison between the two studies. In this set-up, the triceps is positioned in a way that represents full elbow extension. However, distal triceps tendon ruptures can occur in flexed elbows as well. Further, it needs to be noted that solely the isolated triceps tendon was tested in the present biomechanical setup. However, in the in vivo situation, complex forces act on the joint, which also involve other anatomical structures such as the collateral ligaments. Nevertheless, the chosen biomechanical set-up allowed for even tension of all triceps tendon fibers and allowed for comparison with previously published studies. For better comparison, only the rupture mechanism of the distal triceps tendon was to be examined and two specimens in which a bony avulsion occurred were excluded in pilot testing. Although bony avulsions do occur, studies have shown that osseous fragments in distal triceps tendon ruptures correspond more closely to enthesophytes than to osseous fragments from the olecranon process [25]. Lastly, the ruptures were filmed and visually evaluated rather than recorded by a motion-tracking device, which might have been less error-prone.

## Conclusion

In summary, partial distal triceps tendon ruptures show a lower, but not significantly different ultimate load to failure than intact tendons, as well as a characteristic rupturing pattern with primary tearing of the superficial and lateral tendon fibers. These findings suggest that partial ruptures could be treated more conservatively than previously practiced. In addition, in diagnosing distal triceps tendon lesions, particular attention should be paid to the superficial and lateral tendon parts in order to not overlook partial ruptures.

## Data Availability

Research was performed at Technical University of Munich, Germany, in the Department for Orthopaedic Sports Medicine and GelenkPunkt, Austria. The datasets used and analyzed during the current study are available from the corresponding author on reasonable request.

## Abbreviations

Et al: Et alii / et aliae
Mm: Millimeter
N: Newton
LTFmax: Maximum load to failure
Q: Quartile
